# Particulate air pollution and health inequalities: a Europe-wide ecological analysis

**DOI:** 10.1186/1476-072X-12-34

**Published:** 2013-07-16

**Authors:** Elizabeth A Richardson, Jamie Pearce, Helena Tunstall, Richard Mitchell, Niamh K Shortt

**Affiliations:** 1 Centre for Research on Environment, Society and Health (CRESH), School of GeoSciences, University of Edinburgh, Edinburgh EH8 9XP, UK; 2 Centre for Research on Environment, Society and Health (CRESH), Institute of Health and Wellbeing, University of Glasgow, Glasgow G12 8RZ, UK

**Keywords:** Air pollution, Health inequalities, Mortality, Europe, Particulate matter, NUTS regions, Exposure, Susceptibility

## Abstract

**Background:**

Environmental disparities may underlie the unequal distribution of health across socioeconomic groups. However, this assertion has not been tested across a range of countries: an important knowledge gap for a transboundary health issue such as air pollution. We consider whether populations of low-income European regions were a) exposed to disproportionately high levels of particulate air pollution (PM_10_) and/or b) disproportionately susceptible to pollution-related mortality effects.

**Methods:**

Europe-wide gridded PM_10_ and population distribution data were used to calculate population-weighted average PM_10_ concentrations for 268 sub-national regions (NUTS level 2 regions) for the period 2004–2008. The data were mapped, and patterning by mean household income was assessed statistically. Ordinary least squares regression was used to model the association between PM_10_ and cause-specific mortality, after adjusting for regional-level household income and smoking rates.

**Results:**

Air quality improved for most regions between 2004 and 2008, although large differences between Eastern and Western regions persisted. Across Europe, PM_10_ was correlated with low household income but this association primarily reflected East–West inequalities and was not found when Eastern or Western Europe regions were considered separately. Notably, some of the most polluted regions in Western Europe were also among the richest. PM_10_ was more strongly associated with plausibly-related mortality outcomes in Eastern than Western Europe, presumably because of higher ambient concentrations. Populations of lower-income regions appeared more susceptible to the effects of PM_10_, but only for circulatory disease mortality in Eastern Europe and male respiratory mortality in Western Europe.

**Conclusions:**

Income-related inequalities in exposure to ambient PM_10_ may contribute to Europe-wide mortality inequalities, and to those in Eastern but not Western European regions. We found some evidence that lower-income regions were more susceptible to the health effects of PM_10_.

## Introduction

Groups or places with lower socioeconomic status (SES) typically have substantially poorer health than more advantaged people or areas [1]. Unequal exposure to health-damaging characteristics of the physical environment has been posited as one factor contributing both to this worse health, and to the widening in health inequalities that has been observed in a number of countries [2]. This assertion is consistent with the findings of the WHO Commission on Social Determinants of Health which suggested that the unequal distribution of health was influenced by the circumstances in which people grow, live, work, and age, including their physical environments [3]. Since the 1970s, a substantial body of evidence has demonstrated that socially disadvantaged groups are often exposed to physical environments that are potentially health damaging [4]. Environmental inequalities research often applies the framework of ‘environmental justice’ (EJ) – the fair distribution of environmental goods and bads [2,5].

Despite the compelling claim that unequal exposure to health damaging environments contributes to socioeconomic inequalities in health, this assertion has rarely been tested. Analyses of environment and health relationships often consider area-level social disadvantage only as a potential confounder (e.g., [6]). This approach assumes that environmental health risks are consistent across different social strata. The possibility of effect modification – different risks for different social groups – has been investigated less frequently [7]. Two pathways may be involved: differential exposure arises when populations with low socioeconomic status have more frequent or intense exposure to environmental hazards (i.e., environmental inequality), and differential susceptibility (i.e., effect modification) occurs when disadvantaged populations are more likely to be harmed by exposure to the same level of environmental hazard [8]. There has been little exploration of the pathways linking environmental inequality and health disparities, although the urgent need for such work has been highlighted by a number of researchers [4,9].

The present study responds to these calls to investigate the contribution of environmental inequality to health inequalities at the population level, by exploring differential exposure and susceptibility to air pollution in Europe. Air pollution in Europe is a transboundary issue: it is not only the regions producing the pollution that are exposed to it or suffer its health consequences [10]. Displacement of environmental hazards has been found at regional, national and international scales [11,12]. We therefore examine the geographical distribution of potentially hazardous levels of air pollution across Europe, and investigate whether environmental disparities are associated with population-level health inequalities.

In Europe, the air pollutant causing most deaths is particulate matter with an aerodynamic diameter ≤ 10 μm (PM_10_) [13]. Exposure to PM_10_ has been associated with increased all-cause, respiratory and cardiovascular mortality [14]. This evidence has been used to develop air quality standards for health protection [15,16] although health effects can occur at lower concentrations [14]. Strong socioeconomic gradients have been found for causes of death linked to air pollution, [17,18] with deprived groups consistently suffering worse health.

International and national air quality policies have brought about significant improvements in air quality in Europe, although these improvements have not been spatially uniform [13]. Differential air pollutant exposure by either area or individual SES has been explored in eight Western European countries with inconsistent conclusions: disadvantaged groups were exposed to higher levels of air pollution in some studies, but the reverse was found in other work [19]. Fewer studies have explored differential susceptibility to air pollution by SES, and all have focussed on one or a few cities in single countries [20-24]. These studies consistently found that “irrespective of exposure, subjects of low socio-economic status experience greater health effects of air pollution” [19]; Hence, it is feasible that differential exposure and susceptibility to air pollution may contribute to the continuance of health inequalities in Europe [25]. However, the existing European evidence is limited in scope, resulting in uncertainties about the generalisability of the results to other contexts, and particularly to Eastern Europe. We address this paucity of geographical coverage by undertaking a Europe-wide analysis at the level of sub-national regions, to facilitate comparisons both within and between nations.

We addressed the following research questions:

1. To what extent do potentially health-damaging levels of PM_10_ vary across the regions of Europe?

2. Are regions with lower average household income disproportionately exposed to lower air quality?

3. Are populations of regions with lower average household income disproportionately susceptible to the health effects of lower air quality?

## Methods

We adopted an ecological study design to address our research questions. Such a design enables comparability across multiple nation states and generalisability. Additionally, individual-level data with sufficient Europe-wide coverage and sample sizes were not available. We used ambient PM_10_ concentrations within each region as an indicator of population ‘exposure’, and used regional differences in associations between PM_10_ and mortality to indicate ‘susceptibility’.

### Spatial units

We sought units that could be compared between countries and for which appropriate datasets were available. The Nomenclature of Territorial Units for Statistics (NUTS) geography was designed to provide units for statistical comparisons. We selected level 2 of the 2006 version of this geography (NUTS2 regions hereafter) which guidance states should contain between 0.8 and 3 million people.

### Air pollution data

We obtained annual PM_10_ data for 2004 to 2008 from the European Environment Agency’s (EEA) public air quality database ‘AirBase’. As health impacts can vary with exposure time, we obtained indicators of short- and long-term exposure: the 36th highest *daily* mean concentration (μg.m^-3^) and the *annual* average concentration (μg.m^-3^), respectively. The AirBase data had been interpolated from air pollution monitoring data from the European Air Quality Monitoring Network (sites that meet specified data quality criteria), supplemented with altitude, meteorological and concentration modelling data, and were referenced to a 10 × 10 km grid [26]. These interpolated data, developed at the European scale, may differ slightly from within-country assessments.

As populations and particulate pollution tend to be spatially correlated we calculated population-weighted regional averages to reflect the average air quality experienced by the population. This approach weighed pollutant concentrations for more populated parts of each region more heavily than those for sparsely populated places. This prevented an underestimation of PM_10_ concentration if a region had, for example, large areas of unpopulated land. First, the 2006 1 km^2^ population distribution grid for Europe [27] was aggregated to give population counts for 10 × 10 km grid cells that were coincident with the air pollution dataset. Second, the PM_10_ concentration for each grid cell was extracted from the AirBase dataset. Third, the population-weighted average concentration for each region was calculated using the following equation:

$$P_{r}=\frac{\sum_{i=1}^{n_{c}}\left(P_{i}\times \mathrm{po}p_{i}\right)}{\sum_{i=1}^{n_{c}}\left(\mathrm{po}p_{i}\right)}$$

In this equation *P*_*r*_ is the population-weighted PM_10_ concentration for NUTS2 region *r*, *P*_*i*_ is the concentration in the *i*^th^ grid cell within region *r*, *pop*_*i*_ is the population within the *i*^th^ grid cell, and *n*_*c*_ is the total number of grid cells within that region. If any grid cell was split between two or more regions, the cell’s population was divided on the basis of land area (e.g., a region accounting for 75% of the land area of a grid cell would receive 75% of that cell’s population).

### Socioeconomic data

We used average primary household income for private households 2004 to 2008 to measure regional socioeconomic status [28]. Primary household income is the balance generated directly from market transactions – salaries, other income, interest, rent and mortgage payments – before the state’s benefits and taxes are included. Household income has been used as an indicator of SES in health analyses in a wide range of European countries [29]. Average primary household income is estimated using Purchasing Power Consumption Standard units (PPCS) per capita, allowing for meaningful comparison between countries.

### Health data

We selected three causes of death with a plausible aetiological link with PM_10_ – respiratory disease, circulatory disease and all causes – and one with no plausible link, chronic liver disease, for comparison. Age-standardised sex-specific premature (age < 65 y) mortality rates for all causes (International Classification of Disease (ICD) 10 A00-Y89 excluding S00-T98), respiratory diseases (ICD10 J00-J99), circulatory diseases (ICD10 I00-I99), and chronic liver disease (ICD10 K70, K73, K74) were obtained for NUTS2 regions [28]. Three-year moving average rates, standardised to the European standard population, were acquired for 2004–2006, the most recent averaging period with data for most regions. There was however insufficient temporal coverage to investigate trends over time. Separate male and female mortality rates were obtained because sex differences in exposure have been found in other studies [30]. To account for the potentially confounding influence of smoking rate differences between regions [6] we obtained country-level smoking rate estimates derived from the national Health Interview Surveys (2002 collection round) [31].

### Data availability

Air pollution and population data were available for 268 regions of 31 countries between 2004 and 2008 (Austria, Belgium, Bulgaria, Croatia, Czech Republic, Denmark, Estonia, Finland, France, Germany, Hungary, Iceland, Ireland, Italy, Latvia, Liechtenstein, Lithuania, Luxembourg, Macedonia, Malta, Netherlands, Norway, Poland, Portugal, Romania, Slovakia, Slovenia, Spain, Sweden, Switzerland, and United Kingdom). Continuous household income data (2004 to 2008) were not available for ten of these countries, reducing the SES analysis to 235 regions in 12 Western European and 9 Eastern European countries. The regional mortality data (average 2004–2006) were available for 210 regions in 17 countries (161 from the Western European countries of Austria, Finland, France, Germany, Ireland, Netherlands, Norway, Portugal, Spain, Sweden, and UK; and 49 from the Eastern European countries Bulgaria, Czech Republic, Hungary, Poland, Romania, and Slovakia).

As in previous work on health inequalities across Europe [25,32] we excluded certain non-mainland NUTS2 regions that were either atypical of their countries or had very small populations and missing or unreliable data: Åland, Finland; Ceuta, Melilla and the Canary Islands, Spain; French overseas territories; and Madeira and the Azores, Portugal.

### Analyses

The analyses were undertaken in three stages. First, we assessed the spatial and temporal variation in PM_10_ concentrations across NUTS2 regions by mapping them in ArcMap 9.3.1 (ESRI, Redlands, CA). Second, in order to assess variability in pollution according to area-level SES, mean concentrations were calculated for regions grouped into quintiles by their average household income in each year. Summary statistics and correlations between SES and PM_10_ concentrations were calculated using the statistical software Stata/IC 11.0 (StataCorp, College Station, TX). Finally, the relationship between air pollution and health was assessed using ordinary least-squares (OLS) regression analyses to model the relationship between PM_10_ concentrations and regional mortality rates. Models stratified by household income tertiles were run to test whether SES modified the relationship between regional air pollution and health – i.e., disproportionate susceptibility – and the Wald test was used as a formal test for interaction. Pollutant concentrations and household income data for the start year, 2004, were used in these models as proxies for conditions across 2004–2006. Country-level smoking rate estimates were included in all models as continuous percentages.

We investigated spatial autocorrelation in the OLS model residuals, because if the observations are not independent of each other this can lead to artificially small standard errors and false-positive conclusions [33]. We used the GeoDa software [34] to run models corrected for spatial autocorrelation but the results were not substantively different so are not presented here.

## Results

The characteristics of the regions in the study are summarised in Table 1. The short- and long-term PM_10_ measures were highly correlated each year (*r* > 0.97), and analyses revealed virtually identical patterns, hence only results for annual average PM_10_ are presented.

**Table 1:** Demographic, socioeconomic, environmental and health characteristics of the NUTS2 regions in the study in 2004

**Variable**	**n**	**Mean**	**Standard deviation**	**Minimum**	**Maximum**
Population	268	1,848,263	1,462,705	26,347	11,350,290
Area (km^2^)	268	17,552	21,094	160	154,191
Population density (per km^2^)	268	346	835	3	9142
PM_10_: population-weighted annual average (μg.m^-3^)	268	22.3	7.6	2.7	48.5
PM_10_: population-weighted 36th highest daily mean (μg.m^-3^)	268	37.1	13.1	3.4	82.7
Mean household income (PPCS)	235	14,689	5,389	2,736	29,707
Mean smoking rate (%, country level)	29	32.0	1.18	18.6	45.1
Premature mortality rate (per 100,000, 2004–2006)					
All-cause male	210	305.3	124.2	103.2	706.6
All-cause female	210	145.6	40.9	78.3	279.5
Circulatory disease male	210	79.6	52.7	24.2	274.8
Circulatory disease female	210	27.2	19.5	7.8	104.3
Respiratory disease male	210	11.8	7.3	2.8	47.2
Respiratory disease female	210	6.0	3.4	1.5	20.4
Chronic liver disease male	210	16.5	12.9	1.8	80.6
Chronic liver disease female	210	5.9	4.6	0.6	26.2

Abbreviations: *NUTS2* Nomenclature of territorial units for statistics level 2, *PM*_*10*_ Particulate matter with an aerodynamic diameter ≤ 10 μm, *PPCS* Purchasing power consumption standard.

### Q1. How do potentially health-damaging levels of PM_10_ vary across the regions of Europe?

In order to identify ‘potentially health-damaging’ levels of particulate pollution we applied the EU and the World Health Organization (WHO) health standards. The EU Air Quality Directive mandates that annual average PM_10_ should not exceed 40 μg.m^-3^, [15] whereas the WHO recommends a lower target of 20 μg.m^-3^ to significantly reduce health risks [16]. It should be noted that our PM_10_ variable was based on interpolated data produced for use at the European scale, hence may give results that differ from national assessments. Additionally, national compliance with the EU Air Quality Directive is assessed within reporting zones that are often smaller than NUTS2 regions.

**Figure 1:**
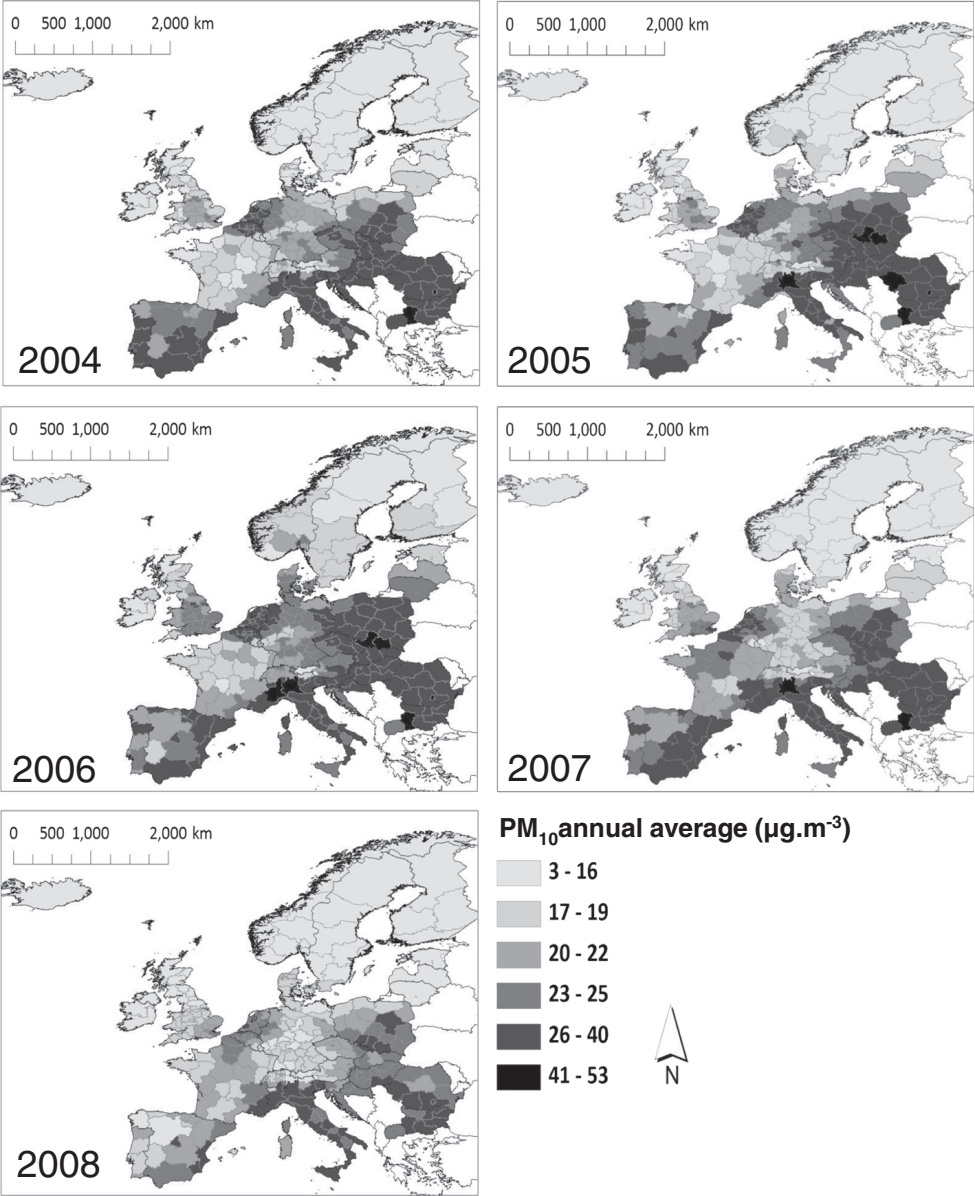
**Regional population-weighted average concentrations of annual average PM**_**10 **_**(μg.m**^**-3**^**) between 2004 and 2008.** Data sources: NUTS2 and country boundary data: GISCO [42]; PM_10_: derived from EEA AirBase data [26].

Throughout the study period PM_10_ concentrations were greatest in the regions of Southern and Eastern Europe, although by 2008 the particulate pollution in these areas was markedly reduced (Figure 1). Breach of the EU’s 40 μg.m^-3^ threshold was rare: this occurred in a maximum of 3% of the regions (*n* = 7) in any one year, and in none in 2008. However in 2006, the most polluted year between 2004–2008, 86% of Western European and 98% of Eastern European regions exceeded the WHO guideline.

### Q2. Are regions with lower average household income disproportionately exposed to lower air quality?

There were significant negative correlations between household income and pollution across Europe (Table 2), with lower-income regions experiencing higher levels of PM_10_. In each year the Europe-wide lowest-income quintile of regions experienced higher PM_10_ concentrations than all other regions, and significantly higher values than the Europe-wide average (Table 2). Approximately 90% of the regions in this quintile were Eastern European. The two highest-income quintiles also tended to have higher PM_10_ values than the overall average, while regions with an intermediate level of income experienced the lowest values.

**Table 2:** **The relationship between regional average household income and population-weighted annual average PM**_**10 **_**(μg.m**^**-3**^**), 2004–2008**

	**(a) Correlation coefficients**	**(b) Pollutant means (μg.m**^**-3**^**, CI)**						
			**by household income quintile (year specific)**					***Ratio Q1:Q5***
		**All regions**	**Q1 (lowest)**	**Q2**	**Q3**	**Q4**	**Q5 (highest)**	
**Whole sample**								
n regions	235	235	47	47	47	47	47	
2004	−0.25**	21.7 (20.7 to 22.7)	27.5 (25.4 to 29.5)^£^	21.4 (19.0 to 23.7)	18.7 (17.3 to 20.1)^$^	22.2 (20.5 to 23.9)	23.1 (21.3 to 24.9)	*1.2*
2005	−0.35***	22.9 (21.9 to 24.0)	30.4 (28.3 to 32.6)^£^	21.7 (19.8 to 23.6)	19.1 (17.6 to 20.6)^$^	23.0 (21.2 to 24.8)	23.0 (21.3 to 24.7)	*1.3*
2006	−0.33***	24.7 (23.7 to 25.6)	31.4 (29.4 to 33.4)^£^	23.8 (22.1 to 25.5)	20.7 (19.1 to 22.3)^$^	25.5 (23.9 to 27.2)	24.7 (23.1 to 26.3)	*1.3*
2007	−0.20**	22.1 (21.3 to 22.9)	25.9 (24.3 to 27.6)^£^	22.1 (20.5 to 23.6)	20.2 (18.9 to 21.6)	22.7 (20.9 to 24.6)	22.5 (20.9 to 24.1)	*1.2*
2008	−0.14*	19.4 (18.8 to 20.0)	22.3 (21.0 to 23.5)^£^	19.4 (18.0 to 20.9)	18.3 (17.2 to 19.4)	20.0 (18.6 to 21.4)	20.2 (18.9 to 21.6)	*1.1*
*2004 to 2008 change (% 2004)*		*−10.7*	*−18.9*	*−9.0*	*−2.1*	*−10.1*	*−12.5*	
**Western Europe**								
n regions	187	187	38	37	38	37	37	
2004	0.10	21.2 (20.3 to 22.1)	21.9 (19.6 to 24.2)	18.0 (16.0 to 19.9)^$^	20.8 (19.1 to 22.5)	22.2 (20.1 to 24.3)	23.1 (21.1 to 25.2)	*0.9*
2005	0.13*	21.5 (20.7 to 22.4)	21.5 (19.5 to 23.5)	18.8 (17.0 to 20.5)^$^	20.9 (19.2 to 22.6)	23.0 (21.2 to 24.9)	23.6 (21.5 to 25.7)	*0.9*
2006	0.17*	23.5 (22.7 to 24.3)	23.1 (21.4 to 24.8)	20.9 (19.2 to 22.6)^$^	23.4 (21.5 to 25.4)	25.0 (23.1 to 26.9)	25.1 (23.3 to 27.0)	*0.9*
2007	0.07	21.9 (21.1 to 22.7)	21.9 (20.1 to 23.8)	20.3 (18.7 to 21.9)	21.4 (19.5 to 23.2)	22.6 (20.8 to 24.4)	23.1 (21.2 to 25.1)	*0.9*
2008	0.08	19.4 (18.8 to 20.1)	19.6 (17.9 to 21.2)	17.8 (16.6 to 19.0)	19.5 (18.2 to 20.8)	20.0 (18.3 to 21.7)	20.1 (18.6 to 21.7)	*1.0*
*2004 to 2008 change (% 2004)*		*−8.5*	*−10.5*	*−0.8*	*−6.1*	*−10.0*	*−13.0*	
**Eastern Europe**								
n regions	48	48	10	10	9	10	9	
2004	−0.01	27.8 (25.8 to 29.9)	31.8 (28.1 to 35.4)	24.7 (20.2 to 29.1)	22.4 (17.4 to 27.4)	31.7 (25.8 to 37.6)	28.1 (25.6 to 30.6)	*1.1*
2005	−0.04	30.8 (28.7 to 32.9)	34.9 (31.6 to 38.2)	28.8 (23.5 to 34.0)	25.1 (20.6 to 29.5)	34.6 (28.3 to 41.0)	30.0 (27.4 to 32.6)	*1.2*
2006	−0.11	32.0 (30.1 to 33.9)	34.5 (32.3 to 36.7)	32.0 (27.6 to 36.5)	27.8 (23.9 to 31.7)	33.3 (27.0 to 39.7)	31.8 (26.6 to 37.0)	*1.1*
2007	−0.17	25.9 (24.3 to 27.5)	29.5 (26.8 to 32.3)	25.9 (22.3 to 29.5)	21.8 (19.2 to 24.4)	25.9 (21.0 to 30.8)	26.1 (21.5 to 30.8)	*1.1*
2008	0.06	22.4 (21.1 to 23.8)	22.5 (20.5 to 24.5)	24.0 (22.0 to 26.1)	18.4 (14.9 to 21.9)	23.6 (20.2 to 27.0)	23.4 (19.2 to 27.6)	*1.0*
*2004 to 2008 change (% 2004)*		*−19.4*	*−29.1*	*−2.5*	*−17.9*	*−25.6*	*−16.8*	

Results given for the whole sample and the Western and Eastern European subsamples: (a) correlation coefficients; (b) mean values (95% confidence interval, CI) for all regions combined and for household income quintiles.

Abbreviations: *CI* confidence interval, *PM*_*10*_ Particulate matter with an aerodynamic diameter ≤ 10 μm.

Correlation coefficients: *0.001 ≤ *p* < 0.1; **0.0001 ≤ *p* < 0.001; *** *p* < 0.0001.

Pollutant means: ^£^ indicates mean is significantly higher than Europe-wide average (*p* < 0.05), ^$^ indicates significantly lower (*p* < 0.05).

Stratified correlation analyses revealed *positive* relationships between PM_10_ and income within Western Europe (significant in 2005 and 2006): each year the highest-income regions experienced higher average concentrations of PM_10_ than the lowest-income regions. PM_10_ concentrations decreased over time for all quintiles, but improvements were greatest in the highest-income regions.

In Eastern European regions the highest PM_10_ concentrations were experienced by the lowest-income regions, in each year except 2008. However, the lowest concentrations were experienced in middle-income regions which, together with the smaller number of regions, may explain why no significant correlations were found. Eastern European regions experienced the greatest improvement in overall air quality: by 2008 average PM_10_ concentrations for these regions were 19% lower than in 2004 compared with 9% for Western Europe. Pollution levels in the lowest-income Eastern European regions fell by the greatest amount over the period (29%).

Across Europe, the 10% of regions with the highest pollutant values were identified. Eleven of these 23 most PM_10_-polluted regions – from Romania, Hungary and Poland – were also among the 10% with the lowest household income. Four of the most PM_10_-polluted regions – Lombardia and Emilia Romagna from Northern Italy, and Flemish Brabant and Walloon Brabant from Belgium – were among the 10% richest.

### Q3. Are regions with lower average household income disproportionately susceptible to the health effects of lower air quality?

In Europe-wide models PM_10_ was related to elevated risk of chronic liver disease, suggesting residual confounding. However, separate analysis of Western and Eastern European regions revealed no relationship between liver disease and PM_10_ (Table 3). Hence we report on the separate analyses for respiratory and circulatory disease and all-cause mortality. In Western European regions PM_10_ was associated with a small increase in risk of respiratory disease mortality for males but not for females, and for no other cause of death. Against Western European mean prevalence the coefficient equated to a 16% increase in male respiratory disease mortality for each 10 μg.m^-3^ increase in annual average PM_10_. In Eastern Europe PM_10_ was associated with increased risk of circulatory disease and respiratory disease mortality for males and females, and all-cause mortality for females. The relative mortality increase related to a 10 μg.m^-3^ increase in PM_10_ was modest for female all-cause mortality (9% of Eastern European mean prevalence), but was more substantial for circulatory disease (males 17% and females 27%) and respiratory disease (20 and 22%, respectively). For most causes of death significantly associated with PM_10_ the absolute ‘effect’ sizes found were twice as high for males as for females, due to differences in baseline prevalence, although in relative terms the associated increase in female deaths was greater.

**Table 3:** **Regression coefficients (+ 95% confidence intervals) for the relationship between PM**_**10 **_**concentration and cause- and sex-specific age-adjusted mortality rate**

**Cause of death**	**Western Europe**		**Eastern Europe**	
	**Male**	**Female**	**Male**	**Female**
All cause	0.74 (−0.28 to 1.77)	−0.30 (−0.78 to 0.18)	1.33 (−1.86 to 4.53)	1.74 (0.39 to 3.08)*
Circulatory disease	−0.16 (−0.51 to 0.19)	0.06 (−0.08 to 0.20)	2.67 (1.44 to 3.90)***	1.38 (0.80 to 1.96)***
Respiratory disease	0.15 (0.06 to 0.24)**	−0.01 (−0.08 to 0.05)	0.42 (0.17 to 0.67)**	0.19 (0.07 to 0.32)**
Chronic liver disease	0.08 (−0.10 to 0.25)	0.00 (−0.07 to 0.07)	0.68 (−0.04 to 1.40)	0.24 (−0.01 to 0.49)

Models were adjusted for regional-level household income and country-level smoking rate and run separately for Eastern and Western Europe. PM_10_ concentration = 2004 population-weighted annual average (μg.m^-3^). Mortality rates = 3-year average 2004–2006 (deaths per 100,000).

Abbreviations: *PM*_*10*_, Particulate matter with an aerodynamic diameter ≤ 10 μm.

* 0.01 ≤ *p* < 0.05; ** 0.001 ≤ *p* < 0.01; *** *p* < 0.001.

**Figure 2:**
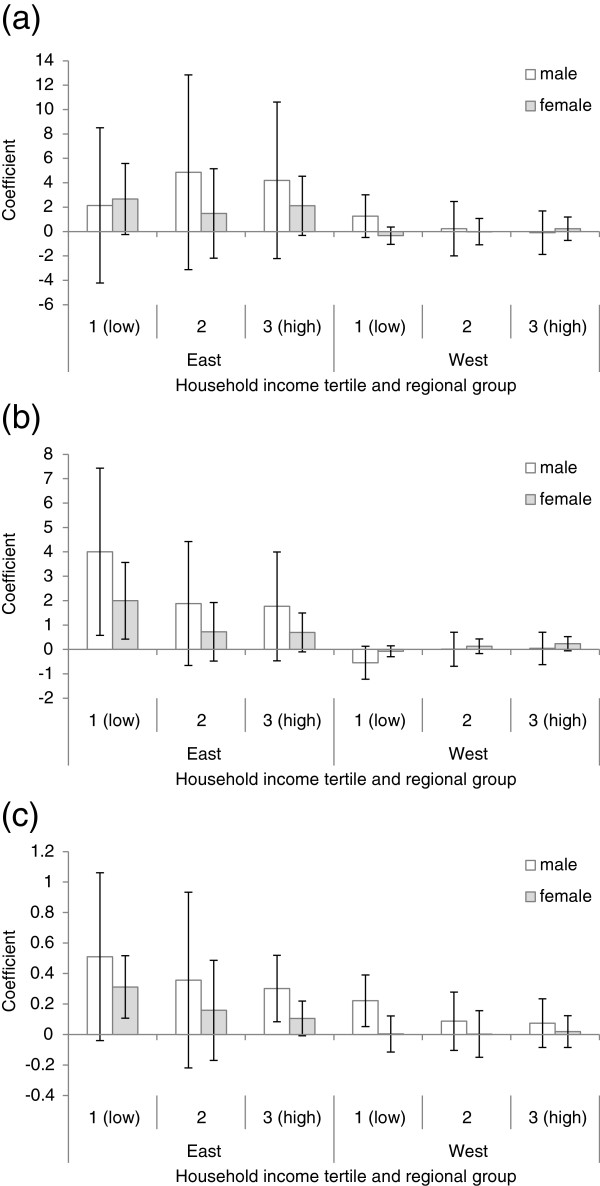
**The relationship between PM**_**10 **_**and (a) all cause, (b) circulatory disease, and (c) respiratory disease mortality, stratified by household income tertiles.** Models were adjusted for household income and were run separately for Eastern and Western Europe. PM_10_ concentration = 2004 population-weighted annual average (μg.m^-3^). Mortality rates = 3-year average 2004–2006 (deaths per 100,000). Error bars indicate 95% confidence intervals.

We assessed whether the relationships between PM_10_ and mortality varied across regions grouped according to average household income (Figure 2). Many of the resulting associations were in the expected direction but lacked statistical significance due to small sample sizes. The lowest-income regions exhibited significantly elevated risks (*p* ≤ 0.03) for male and female circulatory disease mortality in Eastern Europe (*R*^*2*^ = 0.62 and 0.66 respectively) and male respiratory disease mortality in Western Europe (*R*^*2*^ = 0.18). However, no significant interaction effects for household income tertiles in the relationship between PM_10_ and mortality were found.

## Discussion

We investigated whether low income regions in Europe experienced the double jeopardy of exposure to poor air quality as well as social disadvantage. We also considered the associations between PM_10_ and related health outcomes to examine whether low-income areas were disproportionately susceptible to health effects.

Annual average PM_10_ was greatest in the regions of Southern and Eastern Europe, but declined in all regions between 2004 and 2008. Very few regions experienced annual average PM_10_ concentrations higher than those set by the EU Air Quality Directive for the protection of human health, but most exceeded the WHO’s guideline value, indicating the potential for further Europe-wide improvement that would benefit health. Health effects have been shown for PM_10_ concentrations below the EU threshold, hence WHO have recently recommended that the regulations are amended [35].

We found clear evidence of environmental inequality when analysing Europe as a whole. However, the double disadvantage of low income and poor air quality was disproportionately concentrated in Eastern European regions and these were driving the Europe-wide association. Among Western regions only, we observed a positive relationship between income and PM_10_ levels. Such stark differences between associations highlights the importance of scale when addressing these research questions.

The East–West differences in ambient pollution are particularly notable because all countries included, except Norway and Croatia, are subject to the same EU pollution regulations. Eastern European countries were required to meet the EU Air Quality Directive by their accession in 2004 or 2007, although some concessions were made to aid their transition. Latvia, for example, had no system of hazardous waste management until 1995 [36]. But while air quality regulations are being harmonised across Europe, less wealthy Eastern European nations balance these new pressures against those of continuing economic disadvantage [37]. These countries have taken financial advantage of opportunities for international trade, by exporting the products of heavy industry and importing hazardous wastes for disposal [38]. Both types of transaction have the potential for increasing the East-West disparity in environmental quality.

Contrary to expectations, the richest regions were rarely the least polluted; rather the lowest levels of pollution were found among regions with an intermediate level of household income. In Western Europe income and pollution were *positively* correlated: the highest PM_10_ concentrations were consistently found in the highest income regions. High levels of pollution and wealth were co-located in the highly-populated commercial centres of Belgium and the Northern Italian regions involved in high-end automobile and machinery manufacture. In Eastern Europe, although the lowest-income regions were the most polluted in most years, concentrations were lowest in the middle-income regions, hence there was no overall income gradient in air quality. Dawson [36] observes that, in the transition economies of Eastern Europe, the economic benefits of polluting activities appear to have outweighed potential environmental quality and health concerns. Our finding of no clear relationship between income and air quality in these regions supports this claim.

The associations between PM_10_ and health also demonstrated an East–West dichotomy. In Western Europe, out of three plausibly-related health outcomes, PM_10_ was only related to increased risk of male respiratory disease mortality. In Eastern European regions we found significantly elevated risks for male and female circulatory and respiratory disease mortality, and female all-cause mortality. Air pollution is a major risk factor for respiratory disease, but circulatory disease has a number of more influential risk factors: smoking, physical inactivity, unhealthy diet, overweight and high blood pressure. Even though we adjusted for smoking rate differences, albeit crudely, it is possible that the effects of this and other determinants dwarf the contribution of air pollution to circulatory disease mortality rates in Western Europe, with its relatively low levels of pollution. In Eastern Europe, the PM_10_ concentrations are perhaps high enough to contribute to population-level circulatory disease rates. Additionally, as respiratory diseases contribute less to overall mortality than circulatory diseases, the finding of no relationship with all-cause mortality in Western Europe is unsurprising. The significant association with male but not female respiratory disease mortality in Western Europe may be attributable to differential exposure patterns: individual exposure to and inhalation of air pollution is dependent on mobility, time spent indoors and outdoors, and the level of physical activity being undertaken [30]. It may alternatively indicate residual confounding by SES, as male deaths are likely to be more strongly associated with regional income (as seen in Figure 2).

Other work suggests that the relationship between air pollution and health does not differ between Eastern and Western Europe [39]. Both the minimum and maximum annual average concentrations were ~10 μg.m^-3^ higher in the Eastern than the Western European regions in our study (13 to 49 μg.m^-3^ in the East and 3 to 40 μg.m^-3^ in the West in 2004). Detectable health associations were found for the concentrations spanned by the higher range, including for circulatory disease. We suggest that air pollution is a more important risk factor for circulatory diseases at the concentrations found in Eastern than in Western Europe.

We examined whether poorer regional populations were disproportionately susceptible to the health effects of ambient air quality, as indicated in other studies [8]. If the elevated risk among lower-income regions was attributable to PM_10_ we might again expect these effects to be found for respiratory disease mortality ahead of circulatory disease mortality. However, for respiratory disease, increased susceptibility within lower-income regions was only found for males in Western Europe. In Eastern Europe, populations in the lowest-income regions had disproportionately elevated risks of male and female circulatory disease, but not male respiratory disease, for an equivalent increase in annual average PM_10_. Although we adjusted for regional income and smoking within each income grouping, it is possible that other circulatory disease risk factors which are also socially patterned – such as diet or physical activity – may have contributed to the disproportionate ‘effect’. While some high-income regions also experienced high pollution, mortality in these regions was not related to PM_10_ concentrations.

Our study had limitations. First, the characterisation of ‘exposure’ to air pollutants is a clear problem for ecological analysis. Our air pollution measures captured ‘typical’ ambient air quality for each region, but this does not necessarily equate with the exposure experienced by the population. We did not consider indoor exposures or individual activity spaces. Nonetheless, our population-weighting technique aimed to reflect typical ambient conditions where the population was concentrated, hence it provides some improvement over discrete monitoring points or area averages. Second, and related, the ecological fallacy is a potential concern in an analysis of regional-level associations. Hence our findings cannot be assumed to translate into air pollution responses at the individual level. Future work could combine individual- and area-level data to explore these relationships. Third, as a cross-sectional study we cannot draw causal inference from this analysis – a key limitation is our inability to account for the accumulation of exposure across the lifecourse, particularly if exposure had occurred in regions other than the region of residence in 2004. Fourth, unmeasured regional variations may have affected our results. The strong positive associations between mortality rates and PM_10_ found among the poorest regions in Europe may reflect the impact upon health of other unmeasured aspects of socio-economic status (e.g., health behaviours). Also, our inclusion of a single environmental factor did not recognise the simultaneous multiple exposures experienced by populations [9]. Finally, there are additional implications of our use of such large units of analysis, including the Modifiable Areal Unit Problem (MAUP) [40]. Other researchers have found that opposing results can be obtained by analysing the same data at different levels of aggregation, [41] hence our NUTS2-level analysis should be interpreted in this context. We used NUTS2 regions in order to maximise geographical and temporal coverage: if it had been possible to complete our analyses for a smaller geography it is likely that we would have found wider inequalities, largely due to the greater range in pollution and SES values.

## Conclusions

The study confirmed that, while air quality is improving, most regions experience annual average PM_10_ concentrations that exceed those recommended by the WHO, and that stark East–West differences persist. The Europe-wide finding of higher pollution for lower-income regions was not borne out in separate Eastern and Western Europe analyses. Most notably, richer Western European regions tended to experience higher pollution levels than lower-income regions, owing to their wealth-generating industry and commerce.

Ambient particulate air pollution levels were more strongly related to mortality outcomes in Eastern than Western Europe, perhaps reflecting the higher concentrations in Eastern regions. The effects of air pollution may also be dwarfed by those of other non-communicable disease risk factors in Western Europe. We found some indication that the populations of lower-income regions were more susceptible to the health effects of PM_10_, but the evidence varied between Eastern and Western Europe, and between mortality outcomes. Hence, understanding air pollution and its effects may assist our understanding of the geography of health inequalities within Europe, although the relationships may depend on the geographical scope of enquiry.

## Abbreviations

EEA: European Environment Agency; EJ: Environmental Justice; ERC: European Research Council; EU: European Union; ICD: International Classification of Disease; MAUP: Modifiable Areal Unit Problem; NUTS: Nomenclature of Territorial Units for Statistics; OLS: Ordinary Least-Squares regression; PM10: Particulate Matter with an aerodynamic diameter ≤ 10 μm; PPCS: Purchasing Power Consumption Standard; SES: Socioeconomic Status; UK: United Kingdom; WHO: World Health Organization.

## Competing interests

The authors declare they have no conflicts of interest.

## Authors’ contributions

ER acquired, processed and analysed the data and drafted the manuscript. RM, JP and NS conceived of the study, and all authors participated in study design, interpretation and manuscript revision. All authors read and approved the final manuscript.

## References

[B1] Singh GK Siahpush M Widening socioeconomic inequalities in US life expectancy, 1980–2000 Int J Epidemiol 2006 35 4 969 979 16684899 10.1093/ije/dyl083

[B2] Pearce J Richardson EA Mitchell R Shortt NK Environmental justice and health: the implications of the socio-spatial distribution of multiple environmental deprivation for health inequalities in the United Kingdom Trans Inst Br Geogr 2010 35 4 522 539

[B3] Commission on Social Determinants of Health Closing the gap in a generation 2008 Geneva: World Health Organization

[B4] Brulle RJ Pellow DN Environmental justice: human health and environmental inequalities Annu Rev Public Health 2006 27 103 124 16533111 10.1146/annurev.publhealth.27.021405.102124

[B5] Bullard RD Solid wastes sites and the black Houston community Sociol Inq 1983 53 273 288 11635985 10.1111/j.1475-682x.1983.tb00037.x

[B6] Knox EG Atmospheric pollutants and mortalities in English local authority areas J Epidemiol Community Health 2008 62 5 442 447 18413458 10.1136/jech.2007.065862

[B7] Martuzzi M Mitis F Forastiere F Inequalities, inequities, environmental justice in waste management and health Eur J Public Health 2010 20 1 21 26 20061348 10.1093/eurpub/ckp216

[B8] Laurent O Bard D Filleul L Segala C Effect of socioeconomic status on the relationship between atmospheric pollution and mortality J Epidemiol Community Health 2007 61 8 665 675 17630363 10.1136/jech.2006.053611 PMC2652988

[B9] Evans GW Kantrowitz E Socioeconomic status and health: the potential role of environmental risk exposure Annu Rev Public Health 2002 23 303 331 11910065 10.1146/annurev.publhealth.23.112001.112349

[B10] World Health Organization Regional Office for Europe Health risks of ozone from long-range transboundary air pollution 2008 Copenhagen: WHO-Europe

[B11] Carruthers DV The globalization of environmental justice: lessons from the US-Mexico border Soc Nat Resour 2008 21 7 556 568

[B12] Smith D Blowers A Blowers A, Clark M, Smith D Here today, there tomorrow: the politics of hazardous waste transport and disposal Waste location: spatial aspects of waste management, hazards and disposal 1992 New York: Routledge 208 226

[B13] European Environment Agency Air quality in Europe - 2012 report 2012 Copenhagen: EEA

[B14] Anderson HR Atkinson RW Peacock JL Marston L Konstantinou K Meta-analysis of time-series studies and panel studies of particulate matter (PM) and ozone (O3). Report of a WHO task group 2004 Geneva: World Health Organization

[B15] European Parliament and Council Directive 2008/50/EC of the European Parliament and of the Council of 21 May 2008 on ambient air quality and cleaner air for Europe 2008 Brussels: European Parliament and Council

[B16] World Health Organization Air Quality Guidelines: Global Update 2005 2005 Geneva: World Health Organization

[B17] Huisman M Kunst AE Bopp M Borgan J-K Borrell C Costa G Deboosere P Gadeyne S Glickman M Marinacci C Educational inequalities in cause-specific mortality in middle-aged and older men and women in eight western European populations Lancet 2005 365 9458 493 500 15705459 10.1016/S0140-6736(05)17867-2

[B18] Mackenbach JP Bos V Andersen O Cardano M Costa G Harding S Reid A Hemstrom O Valkonen T Kunst AE Widening socioeconomic inequalities in mortality in six Western European countries Int J Epidemiol 2003 32 5 830 837 14559760 10.1093/ije/dyg209

[B19] Deguen SV Zmirou-Navier D Social inequalities resulting from health risks related to ambient air quality - A European review J Public Health 2010 20 1 27 35 10.1093/eurpub/ckp220 20081212

[B20] Forastiere F Stafoggia M Tasco C Picciotto S Agabiti N Cesaroni G Perucci CA Socioeconomic status, particulate air pollution, and daily mortality: differential exposure or differential susceptibility Am J Ind Med 2007 50 3 208 216 16847936 10.1002/ajim.20368

[B21] Filleul L Rondeau V Cantagrel A Dartigues JF Tessier JF Do subject characteristics modify the effects of particulate air pollution on daily mortality among the elderly? J Occup Environ Med 2004 46 11 1115 1122 15534498 10.1097/01.jom.0000144998.82543.9d

[B22] Laurent O Pedrono G Filleul L Segala C Lefranc A Schillinger C Riviere E Bard D Influence of socioeconomic deprivation on the relation between Air pollution and beta-agonist sales for asthma Chest 2009 135 3 717 723 19017882 10.1378/chest.08-1604

[B23] Havard S Pedrono G Deguen S Schillinger C Segala C Riviere E Arveiler D Bard D Air pollution and myocardial infarction-a small-area case crossover in Strasbourg, France influence of individual and area characteristics Epidemiol 2008 19 6 S197 S197

[B24] Wojtyniak B Stokwiszewski J Rabczenko D Air pollution and low birth weight in Polish urban population Epidemiol 2001 12 4 S64 S64

[B25] Richardson EA Mitchell R Pearce J Shortt NK Tunstall H Have regional health inequalities in life expectancy widened within the European Union between 1991 and 2008? Eur J Public Healthin press 10.1093/eurpub/ckt084 23813717

[B26] Horálek J Denby B de Smet PAM de Leeuw FAAM Kurfürst P Swart R van Noije T Spatial mapping of air quality for European scale assessment. ETC./ACC Technical paper No 2006/6 2007 Bilthoven: European Topic Centre on Air and Climate Change Accessed 22.10.2012 from http://acm.eionet.europa.eu/docs/ETCACC_TechnPaper_2006_6_Spat_AQ.pdf

[B27] Eurostat JRC GEOSTAT GEOSTAT 1km2 population grid 2006 Luxembourg: European Commission

[B28] Eurostat New Cronos (Data downloaded: 25 April 2012) 2012 Manchester: ESDS International, University of Manchester

[B29] Mackenbach JP Stirbu I Roskam AJR Schaap MM Menvielle G Leinsalu M Kunst AE European Union Working Grpoups Socioeconomic inequalities in health in 22 European countries N Engl J Med 2008 358 23 2468 2481 18525043 10.1056/NEJMsa0707519

[B30] Abbey DE Burchette RJ Knutsen SF McDonnell WF Lebowitz MD Enright PL Long-term particulate and other air pollutants and lung function in nonsmokers Am J Respir Crit Care Med 1998 158 1 289 298 9655742 10.1164/ajrccm.158.1.9710101

[B31] Eurostat Health status: indicators from the national Health Interview Surveys (collection round 2002) 2004 Luxembourg: Eurostat

[B32] Shaw M Orford S Brimblecombe N Dorling D Widening inequality in mortality between 160 regions of 15 European countries in the early 1990s Soc Sci Med 2000 50 1047 1058 10714926 10.1016/s0277-9536(99)00354-8

[B33] Bowen W An analytical review of environmental justice research: what do we really know? Environ Manage 2002 29 1 3 15 11740620 10.1007/s00267-001-0037-8

[B34] Anselin L Exploring spatial data with GeoDa™: a workbook 2005 Center for Spatially Integrated Social Science: Urbana, IL Accessed 22.10.2012 from https://geodacenter.asu.edu/system/files/geodaworkbook.pdf

[B35] World Health Organization Review of evidence on health aspects of air pollution - REVIHAAP. First Results 2013 Bonn: WHO Regional Office for Europe 27195369

[B36] Dawson JI Latvia’s Russian minority: balancing the imperatives of regional development and environmental justice Polit Geogr 2001 20 7 787 815

[B37] Hudson R The costs of globalization: producing new forms of risk to health and well-being Risk Manage 2009 11 13 29

[B38] Steger T Making the Case for Environmental Justice in Central & Eastern Europe 2007 Budapest: CEU Centre for Environmental Policy and Law

[B39] Bobak M Feachem RGA Air pollution and mortality in central and eastern Europe Eur J Public Health 1995 5 2 82 86

[B40] Openshaw S The Modifiable Areal Unit Problem 1984 Norwich: Geo Books

[B41] Baden BM Noonan DS Turaga RMR Scales of justice: Is there a geographic bias in environmental equity analysis? J Env Plan Manage 2007 50 2 163 185

[B42] Eurostat GISCO Administrative boundaries of NUTS 2006 2006 Luxembourg: Eurostat GISCO - European Commission http://epp.eurostat.ec.europa.eu/cache/GISCO/geodatafiles/NUTS_2006_03M_SH.zip

